# Clinical Characteristics of Neovascular Age-Related Macular Degeneration without Typical Drusen

**DOI:** 10.1155/2021/6683532

**Published:** 2021-04-27

**Authors:** Hiroyuki Kamao, Katsutoshi Goto, Kento Matsuno, Kenichi Mizukawa, Atsushi Miki, Junichi Kiryu

**Affiliations:** ^1^Department of Ophthalmology, Kawasaki Medical School, 577 Matsushima Kurashiki, Okayama 701-0114, Japan; ^2^Shirai Eye Hospital, 1339 Takasecho Kamitakase, Mitoyo, Kagawa 767-0001, Japan

## Abstract

**Purpose:**

To evaluate the clinical characteristics of neovascular age-related macular degeneration (nAMD) patients without typical drusen.

**Methods:**

We retrospectively studied 165 eyes in 165 patients with treatment-naïve nAMD, including typical AMD and polypoidal choroidal vasculopathy (PCV). According to the fellow eye condition, the patients were divided into nAMD with and without typical drusen groups. Eyes with soft drusen or subretinal drusenoid deposits were classified into the nAMD with the typical drusen group. Smoking status and diagnoses of hypertension and diabetes were identified from hospital records and patient recall. We assessed best-corrected visual acuity (BCVA), central retinal thickness (CRT) at the fovea, subfoveal choroidal thickness (SFCT), and the number of injections received.

**Results:**

The nAMD without typical drusen group was significantly younger (77.9 ± 7.6 vs. 71.8 ± 8.3, *P* < 0.001) and had thicker SFCT at baseline (207.9 ± 99.5 vs. 260.1 ± 113.2 *μ*m, *P*=0.007) and a higher proportion of PCV (30.6 vs. 63.1%, *P* < 0.001). The proportion of ever-smokers was significantly higher in the nAMD without typical drusen group (54.8 vs. 70.9%, *P*=0.036). There were no statistically significant differences in the proportion of patients with hypertension or diabetes; BCVA, CRT, or SFCT changes; or the number of injections between the nAMD with and without typical drusen groups.

**Conclusion:**

The clinical features of patients in the nAMD without typical drusen group were almost identical to those of pachychoroid-driven choroidal neovascularization (CNV) patients. The nAMD without typical drusen group had a significantly higher proportion of ever-smokers than the nAMD with typical drusen group. Smoking could be a risk factor for the development of pachychoroid-driven CNV.

## 1. Introduction

“Pachychoroid” is a term introduced by Warrow et al. to systematically define macular diseases with a thick choroid [1] that features choroidal vascular hyperpermeability (CVH) and dilatation of the large choroidal vessels in Haller's layer with attenuation of the choriocapillaris and Sattler's layer. Choroidal abnormalities are associated with progressive retinal pigment epithelium (RPE) dysfunction, followed by choroidal neovascularization (CNV), including pachychoroid neovasculopathy (PNV) [2] and polypoidal choroidal vasculopathy (PCV) [3]. Elderly patients presenting with macular neovascularization are diagnosed with neovascular age-related macular degeneration (nAMD), including PCV, and drusen have been described as retinal precursor lesions of nAMD [4, 5]. However, PCV is a major subtype of nAMD in Asian people, a feature of which is a comparatively low incidence of drusen [6]. Even among Caucasians with a high prevalence of drusen, the incidence of drusen is lower (16.7%) in Caucasian patients with PCV [7]. These observations indicate that the development of nAMD is classified into a drusen-dependent pathway and a pachychoroid-dependent pathway. Previous studies have demonstrated that there are differences in genetic background and clinical features between pachychoroid nAMD and nonpachychoroid nAMD [8–10]; therefore, risk factors for pachychoroid-driven CNV could differ from drusen-driven nAMD. Many observational studies associated with nAMD have identified several modifiable risk factors, such as smoking [11–14], systemic hypertension [14, 15], dyslipidemia [15, 16], and dietary fat consumption [17]. However, the risk factors associated with pachychoroid-driven CNV have not been clarified. Because the choroidal thickness correlated with the patient's age and axial length prevents defining the thickened choroid [18, 19], there is no consensus for the diagnostic criteria of pachychoroid diseases.

Recently, drusen are classified into soft drusen, subretinal drusenoid deposits (SDDs), and pachydrusen [20]. Soft drusen and SDDs are conventional drusen, while pachydrusen reported as drusen associated with pachychoroid, distinguished from the conventional drusen commonly found in AMD [21]. Additionally, pachydrusen did not increase the risk of progression to nAMD [22]. Therefore, pachychoroid diseases are a condition characterized by a lack of typical drusen, including soft drusen or SDDs, and pachychoroid-driven CNV mostly overlaps with nAMD without typical drusen. In the present study, we classified nAMD by the presence or absence of typical drusen and determined the differences in risk factors between nAMD with and without typical drusen to estimate the risk factors for pachychoroid-driven CNV.

## 2. Materials and Methods

### 2.1. Study Design and Participants

We performed this study, which was conducted according to the Declaration of Helsinki principles and registered with the UMIN Clinical Trials Registry (UMIN000023676), with approval from the Kawasaki Medical School Ethics Committee (2543–1). We retrospectively studied 165 eyes of 165 patients with typical AMD (tAMD) and PCV. Patients with retinal angiomatous proliferation (RAP) were excluded because of smaller sample sizes (15 eyes in 10 patients). Data regarding cigarette smoking, hypertension, and diabetes were collected from patient recall or hospital records. We divided the patients into never-smokers and ever-smokers as described in a previous report [23]. All participants received a complete ophthalmologic examination, including measurement of best-corrected visual acuity (BCVA), indirect ophthalmoscopy, slit-lamp biomicroscopy with a noncontact lens, color fundus photography and fundus autofluorescence (Canon CX-1; Canon, Tokyo, Japan), swept-source optical coherence tomography (OCT) (DRI OCT-1 Atlantis; Topcon Corporation, Tokyo, Japan), fluorescein and indocyanine green angiography (HRA-2; Heidelberg Engineering GmbH, Dossenheim, Germany). Visual acuity data were obtained in decimal BCVA and converted to the logarithm of the minimum angle of resolution (logMAR) units for the analysis. The retinal and choroidal thickness were measured by swept-source OCT as described in a previous report [23]. Patients with CNV as a result of high myopia, angioid streaks, hereditary disorders, or uveitis were excluded.

### 2.2. Group Classification

**Figure 1:**
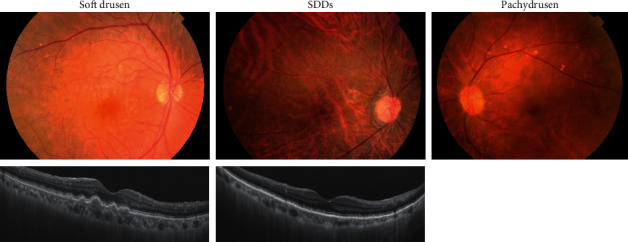
Fundus color photograph and OCT of soft drusen, SDDs, and pachydrusen.

We classified nAMD patients into two groups depending on the condition of the fellow eye as follows: eye with soft drusen or SDDs is nAMD with typical drusen group and eye with no significant drusen and pachydrusen is nAMD without typical drusen group (Figure 1). The type of drusen was determined using fundus color photographs and swept-source OCT according to the criteria presented in a previous study [20]. Nonsignificant drusen included eyes without drusen or eyes with small drusen (size: <63 *μ*m) or a few intermediate drusen (number: <20 lesions, size: <125 *μ*m). Soft drusen included numerous intermediate drusen (number: ≥20 lesions, size: ≥63 *μ*m and <125 *μ*m) or one large druse (size: ≥125 *μ*m), according to the Age-Related Eye Disease Study (AREDS). Eyes with pachydrusen and soft drusen or SDDs were classified into soft drusen or SDDs, respectively.

### 2.3. Treatment and Assessments

All patients were treated with the treat-and-extend basis of intravitreal aflibercept (IVA) between May 2013 and April 2020, followed by one year or longer follow-up. Patients who had received other anti-VEGF agents (bevacizumab, pegaptanib, or bevacizumab) or had undergone verteporfin photodynamic therapy or laser photocoagulation were excluded. For the primary outcome, the mean change in BCVA from baseline to the final visit was compared between the nAMD with and without typical drusen groups. The mean change in retinal and choroidal thickness values at the fovea, duration of follow-up, and the number of injections received were also compared between the two groups for the secondary outcome measures.

### 2.4. Statistical Analysis

The differences between age, BCVA, CRT, SFCT, duration of follow-up, and the number of injections received between the nAMD with and without typical drusen groups were compared by the Mann–Whitney *U* test. The chi-square test was used to compare the differences of proportions in sex, hypertension, diabetes, smoking habits (never- or ever-smokers), AMD subtype, and frequency of severe complication (RPE tear and subretinal hemorrhage) between the nAMD with and without typical drusen groups. Kaplan–Meier analysis was performed to estimate the incidence of nAMD in the fellow eye. A log-rank test was used to analyze the differences in time to the incidence of nAMD between the two groups. The statistical analyses were performed by Ekuseru-Toukei 2012 (Social Survey Research Information Co., Ltd). *P* values <0.05 were considered statistically significant in all analyses, ^*∗*^Mann–Whitney *U* test and †chi-square test.

## 3. Results

**Figure 2:**
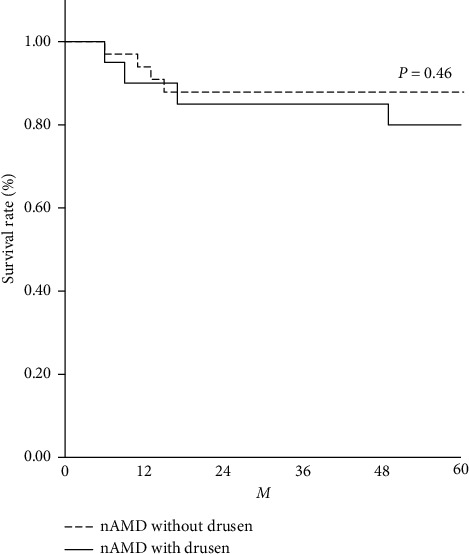
The 5-year incidences of nAMD in the fellow eye.

In total, 165 eyes of 165 patients with nAMD were included. The characteristics of the nAMD patients are shown in Table 1. The mean (±SD) age was 74.1 ± 8.6 (range, 50–94) years. The analysis included 62 patients in the nAMD with typical drusen group (22 women, 40 men; mean age 77.9 ± 7.6 [range, 58–94] years) and 103 patients in the nAMD without typical drusen group (26 women, 77 men; mean age 71.8 ± 8.3 [range, 50–92] years). The two study groups were comparable with regard to the female-to-male ratio; however, the patients were significantly younger in the nAMD without the typical drusen group (*P* < 0.001). The proportions of hypertension, diabetes, and ever-smokers were 47%, 13%, and 55% in the nAMD with typical drusen group and 46%, 22%, and 71% in the nAMD without typical drusen group, respectively. The two study groups were comparable with regard to the proportions of hypertension and diabetes; however, there was a significantly higher proportion of ever-smokers in the nAMD without the typical drusen group (*P* < 0.05). The proportions of tAMD and PCV in the two study groups were 69% and 31% in the nAMD with typical drusen group and 37% and 63% in the nAMD without typical drusen group, respectively; the PCV was significantly higher in the nAMD without typical drusen group. After a follow-up of 5 years, 8 of 53 (15.1%) fellow eyes developed nAMD. The numbers of patients who developed nAMD in the fellow eye were 4 of 20 (20%) and 4 of 33 (12.1%) in the nAMD with and without typical drusen groups, respectively. The Kaplan–Meier curve demonstrated that the incidence of developing nAMD in the fellow eye was not significantly different between the two groups (Figure 2). Similarly, the incidence of developing nAMD in the fellow eye was not significantly different in hypertension, diabetes, smoking habit, or AMD subtypes between the two groups (data not shown).

The outcome of anti-VEGF therapy in the nAMD patients is shown in Table 2 (52 and 81 eyes in the nAMD with and without typical drusen group, resp.). The mean BCVA and CRT were 0.41 ± 0.51 and 317.3 ± 114.8 *μ*m in the nAMD with typical drusen group and 0.30 ± 0.27 and 340.1 ± 178.7 *μ*m in the nAMD without typical drusen group at baseline and 0.52 ± 0.67 and 226.9 ± 70.8 *μ*m and 0.36 ± 0.44 and 239.3 ± 121.9 *μ*m, respectively, at the final visit; the differences were not statistically significant. However, the mean SFCT of the nAMD with and without typical drusen groups were 207.9 ± 99.5 and 260.1 ± 113.2 *μ*m at baseline (*P*=0.007) and 161.3 ± 85.7 and 207.7 ± 97.9 *μ*m at the final visit, respectively (*P*=0.003); the SFCT at the baseline and final visits were significantly thicker in the nAMD without typical drusen group than in the nAMD with typical drusen group. The final BCVA, CRT, and SFCT were improved to a similar extent in both groups of patients. Similarly, there were no significant differences in the duration of patient follow-up and injection frequency of aflibercept received between the nAMD with and without typical drusen groups (5.0 ± 2.4 years, 4.7 ± 3.1 IVA per year vs. 4.9 ± 2.6 years, 4.6 ± 3.0 IVA per year). RPE tear and subretinal hemorrhage occurred in two and three patients in the nAMD with the typical drusen group and two and six patients in the nAMD without the typical drusen group, respectively; these differences were not statistically significant. Besides, we obtained similar results of anti-VEGF treatment efficacy for the nAMD patients, including RAP patients (Table 3).

## 4. Discussion

The present study revealed the differences in clinical characteristics between nAMD with and without typical drusen. The term “pachychoroid” (pachy-[prefix]: thick) was proposed as a term indicating an abnormal increase in choroidal thickness. Several diseases, including central serous chorioretinopathy, pachychoroid pigment epitheliopathy [1], PNV, and PCV, have been found to share this characteristic feature. Miyake et al. evaluated the differences in genetic background and clinical features between PNV and non-PNV and demonstrated that PNV patients were significantly younger and had lower genetic risk scores than non-PNV patients [8]. Additionally, the frequency of genes associated with nAMD (CFH rs800292) in pachychoroid neovasculopathy patients was comparable to that in normal Japanese subjects, suggesting that the risk factors for developing pachychoroid-driven CNV could differ from drusen-driven nAMD. However, the risk factors for the development of pachychoroid-driven CNV have not been clarified. One of the reasons for this is that there is no consensus on the quantitative criteria of pachychoroid. Hence, we focused on the absence of typical drusen including soft drusen or SDDs, which is characteristic of pachychoroid-driven CNV and assessed risk factors for the development of pachychoroid-driven CNV by comparing nAMD with typical drusen and without typical drusen. In the present study, the clinical features of the nAMD without typical drusen group were significantly younger age, a higher proportion of PCV, and a thicker choroid than the nAMD with typical drusen group, indicating that the clinical features of nAMD without typical drusen are almost identical to those of pachychoroid-driven CNV.

As shown in Table 1, the nAMD without typical drusen group had a significantly higher proportion of ever-smokers than the nAMD with typical drusen group, suggesting a difference in the pathogenic process of CNV between nAMD with and without typical drusen. Traditionally, nAMD is a condition characterized by the presence of soft drusen or SDDs [24, 25]. Drusen accumulate extracellular material between the RPE and Bruch's membrane and contain various components, such as cholesterol [26], lipoprotein [27], complement pathway proteins [28, 29], and age-related amyloid deposits [30]. Mutations/polymorphisms in genes coding for alternative complement pathway regulators (factor H and factor H-related proteins) and complement pathway proteins (complement component C2, C3, and factor B) have been identified as genetic factors in the development of AMD [31–34]. Therefore, these components in drusen act as drivers of chronic inflammation that plays an important role in the pathology of AMD. Additionally, population-based cohort studies demonstrated that soft drusen or SDDs are the precursor lesion for AMD [35, 36]. In contrast, little is known about the mechanism of pachychoroid-driven CNV. Although it is unclear whether the attenuation of inner choroidal vessels is a primary pathologic process or a secondary morphological change caused by the mechanical pressure of dilated outer choroidal vessels, ischemic damage to RPE is thought to be caused by inner choroidal attenuation [37, 38]. Several population-based studies [11–13] have shown the risk factors for the nAMD, and cigarette smoking is consistently associated with the development of nAMD. Cigarette smoke contains many different chemicals [39] that cause many chronic diseases, including ischemic heart or brain diseases, through arteriosclerosis induced by accumulating reactive oxygen species. Although the precise mechanisms of CNV formation are still not fully understood, the development of CNV is considered to be caused by impairment of the RPE and Bruch's membrane because of accumulation of oxidative stress and decreased choriocapillaris blood flow. Thus, our results strongly indicate that smoking plays an essential role in the development of pachychoroid-driven CNV caused by attenuation or choriocapillaris. Our results do not mean that smoking is not a risk factor for the development of nAMD with typical drusen. Although it was not shown in the results of this study, the proportion of ever-smokers among healthy elderly subjects was 30% (48/160); the proportion of ever-smokers in the nAMD with typical drusen group was significantly higher than that among healthy subjects. We are thus able to advise not smoking to prevent the incidence of nAMD in the fellow eye.

As Figure 1 shows, the 5-year incidence of nAMD in the fellow eye was 15.1%, and the incidence of developing nAMD in the fellow eye was not significantly different between the nAMD with and without typical drusen groups. The 5-year incidence of nAMD in the Age-Related Eye Disease Study (AREDS), designed to estimate the risk factors of AMD, was 30.8% in unilateral nAMD patients [40]. Meanwhile, a recent study in Asia to investigate the 5-year progression rates of nAMD in the fellow eye was 20.9% [22]. Lee et al. classified the nAMD patients using the new drusen classification and showed that the eye with soft drusen and/or SDDs was a significantly higher risk of the 5-year incidence of nAMD in the fellow eye than the eyes with pachydrusen or no significant drusen. Another study of developing nAMD in fellow eyes in Asian nAMD patients also resulted in that the pachydrusen group had a significantly lower frequency of developing nAMD in the fellow eye than the soft drusen or SDDs groups [41]. Therefore, racial differences may have contributed to the difference between AREDS and our results. Lee et al. examined the difference in the 5-year incidence of nAMD in the fellow eye in nAMD patients categorized by disease subtypes. The incidence in the RAP group was significantly higher than that of the other groups. We excluded RAP patients due to smaller sample sizes (15 eyes in 10 patients). The difference in the drusen classification method and the inclusion of patients with RAP could contribute to the differences in study results.

Various treatment methods, including photodynamic therapy, anti-VEGF therapy, gene therapy, or regeneration therapy [42–47], have been developed for age-related macular degeneration. Anti-VEGF therapy maintains or improves the visual outcome in patients with nAMD and is now used as a first-line treatment in this disease. However, patients who develop recurrence and those who do not respond to the therapy are unlikely to derive much long-term benefit from the therapy, and many patients eventually become dissatisfied with the outcomes of this treatment. Thus, it is crucial to investigate the factors involved in the effectiveness of anti-VEGF therapy. The difference in the efficacy of anti-VEGF therapy between pachychoroid-driven CNV and nonpachychoroid-driven CNV has been shown. Several reports have shown that PNV requires fewer injections than typical nAMD [8, 9]. Miyake et al. demonstrated that PNV had a more extended retreatment-free period after a loading dose than typical AMD. Matsumoto et al. showed that PNV and typical AMD required 13.2 and 13.8 injections in 2 years, respectively, and that the number of injections for PNV was significantly lower. In contrast, several studies reported that nAMD with pachychoroid required more injections than nAMD without pachychoroid [10, 48–51]. The VEGF concentration in PNV was lower than that in nAMD, but it was almost identical to that in the control group, suggesting that VEGF contributes less to the pathogenesis of PNV. Chang et al. reported that PCV patients with pachychoroid showed less response to anti-VEGF therapy than PCV patients without pachychoroid. Hara demonstrated that, among patients with CVH, a clinical feature of pachychoroid, there were more nonresponders to anti-VEGF therapy. In the present study, the visual acuity and retinal and choroidal thickness were improved in both groups, and there was no difference in visual outcome or the number of injections between these two groups. Differences in our results from other studies' results could be caused by the difference in the methods of classification and diagnosis and observation periods. Previous studies on pachychoroid set their own diagnostic criteria, such as subfoveal choroidal thickness ≥200 *μ*m in both eyes. The RPE abnormality or CNV develops at sites of focal dilatation of choroidal vessels even in the eye with standard choroidal thickness; thus, we need to pay attention when comparing studies about the effectiveness of treatment for pachychoroid.

The present study has several limitations. This was a retrospective single-center study, which may have led to selection bias. We classified nAMD by the presence or absence of typical drusen, and nAMD patients with large soft drusen or SDDs were classified into the presence of typical drusen group. Recent reports describing drusen classify them into soft drusen, SDDs, and pachydrusen according to clinical characteristics [21]. Previous studies reported that large soft drusen or SDDs are major risk factors for the development of nAMD [4, 52, 53]. In contrast, pachydrusen, which showed a strong association with pachychoroid [54, 55], did not confer an increased risk of progression to nAMD [22]; thus, we classified nAMD patients with pachydrusen into the absence of the typical drusen group. Additionally, the results obtained in this study are characteristic of nAMD without typical drusen, not identical to those of pachychoroid-driven CNV. The majority of classified nAMD patients in this study had eyes with typical drusen but without pachychoroid features or with pachychoroid features but without typical drusen. However, there were a few eyes with typical drusen and pachychoroid features or without typical drusen and pachychoroid features. AMD patients without typical drusen and pachychoroid features indicated that there might be another driver for the development of nAMD; therefore, the nAMD classification method requires further refinement.

## 5. Conclusions

The clinical features of nAMD without typical drusen were almost identical to those of pachychoroid-driven CNV. The nAMD without typical drusen group had a significantly higher proportion of ever-smokers than the nAMD with typical drusen group. The mechanism of pachychoroid-driven CNV could be caused by ischemic damage to the RPE, as previously reported. Our present findings should heighten awareness about smoking risk among both the general public and health professionals.

## Figures and Tables

**Table 1:** Clinical characteristics of nAMD patients.

	AMD with drusen (*n* = 62)	AMD without drusen (*n* = 103)	*P*
Age (years), mean (SD)	77.9 (7.6)	71.8 (8.3)	<0.001^*∗*^
Gender (female), number (%)	22 (35.5)	26 (25.2)	0.16
Hypertension, number (%)	29 (46.8)	47 (45.6)	0.89
Diabetes, number (%)	8 (12.9)	23 (22.3)	0.13
Smoking habits (ever-smokers), number (%)	34 (54.8)	73 (70.9)	0.036†
AMD subtype, number (%)			<0.001†
PCV	19 (30.6)	65 (63.1)	
Typical AMD	43 (69.4)	38 (36.9)	

**Table 2:** Results of anti-VEGF therapy in nAMD with and without drusen.

	AMD with drusen (*n* = 52)	AMD without drusen (*n* = 81)	*P*
Baseline, mean (SD)			
VA (logMAR)	0.41 (0.51)	0.30 (0.27)	0.80
CRT (*μ*m)	317.3 (114.8)	340.1 (178.7)	0.65
SFCT (*μ*m)	207.9 (99.5)	260.1 (113.2)	0.007^*∗*^
Outcome, mean (SD)			
VA (logMAR)	0.52 (0.67)	0.36 (0.44)	0.28
CRT (*μ*m)	226.9 (70.8)	239.3 (121.9)	0.91
SFCT (*μ*m)	161.3 (85.7)	207.7 (97.9)	0.003^*∗*^
Change, mean (SD)			
VA (logMAR)	0.12 (0.64)	0.05 (0.45)	0.69
CRT (*μ*m)	90.5 (111.7)	100.8 (212.9)	0.98
SFCT (*μ*m)	42.6 (71.0)	49.7 (68.2)	0.54
Follow-up (yrs), mean (SD)	5.0 (2.4)	4.9 (2.6)	0.76
Number of injections (no./yr), mean (SD)	4.7 (3.1)	4.6 (3.0)	0.78
RPE tear, number (%)	2 (3.8)	2 (2.5)	0.65
Subretinal hemorrhage, number (%)	3 (5.8)	6 (7.4)	0.71

**Table 3:** Results of anti-VEGF therapy in nAMD including RAP.

	AMD with drusen (n = 64)	AMD without drusen (n = 84)	*P*
Change, mean (SD)			
VA (logMAR)	0.14 (0.61)	0.04 (0.45)	0.69
CRT (*μ*m)	94.3 (108.6)	104.8 (210.4)	0.98
SFCT (*μ*m)	41.3 (65.3)	46.3 (68.6)	0.54
Number of injection (no./yr), mean (SD)	4.8 (3.7)	4.7 (3.1)	0.78

## Data Availability

The data used to support the findings of this study are restricted by the Kawasaki Medical School Ethics Committee in order to protect patient privacy. The data are available from Hiroyuki, Kamao M.D., Ph.D. [hironeri@med.kawasaki-m.ac.jp] for researchers who meet the criteria for access to confidential data.
